# Compliance with national snakebite treatment guidelines in rural Sri Lankan hospitals: a cluster randomized controlled trial of a brief educational intervention

**DOI:** 10.1186/s12909-023-04375-1

**Published:** 2023-05-27

**Authors:** Seyed Shahmy, Senanayake A. M. Kularatne, Indika B. Gawarammana, Shantha S. Rathnayake, Andrew H. Dawson

**Affiliations:** 1 National Science and Technology Commission of Sri Lanka, Colombo, Sri Lanka; 2 South Asian Clinical Toxicology Research Collaboration, Faculty of Medicine, University of Peradeniya, Peradeniya, Sri Lanka; 3 Department of Medicine, Faculty of Medicine, University of Peradeniya, Peradeniya, Sri Lanka; 4 Central Clinical School, University of Sydney, Sydney, Australia

**Keywords:** Cluster RCT, Educational Intervention, Health care workers, Primary Hospitals, Treatment guidelines, Snakebites, Sri Lanka

## Abstract

**Background:**

Snakebite is a global health problem that predominantly occurs in rural areas. In Sri Lanka, the majority of snakebite patients first present to smaller rural primary hospitals. Improving care delivered at rural hospitals has the potential to reduce morbidity and mortality from snakebites.

**Objective:**

In this study, we evaluated whether an educational intervention would increase compliance with national snakebite treatment guidelines in primary hospitals.

**Methods:**

The hospitals were randomized into educational intervention (*n* = 24) and control groups (*n* = 20). The intervention hospitals received a brief educational intervention based on Sri Lankan Medical Association (SLMA) guidelines on the management of snakebites. Control hospitals had free access to the guidelines but no additional promotion. Four outcomes were assessed: pre- and post-test knowledge at the completion of a one-day workshop of educational intervention (intervention group only); improvement in the quality of the patient’s medical records; appropriateness of transfers to higher hospitals; and quality of overall management graded by a blinded expert. The data was collected over a period of 12 months.

**Results:**

All case notes of snakebite hospital admissions were reviewed. There were 1021 cases in the intervention group hospitals and 1165 cases in the control hospitals. Four hospitals in the intervention group and three hospitals in the control group did not have snakebite admissions and were excluded from the cluster analysis. The absolute quality of care was high in both groups. Post-test knowledge was improved (*p* < 0.0001) following the intervention group’s educational workshop. There was no statistical difference between the two groups in terms of clinical data documentation in hospital notes (scores, *p* = 0.58) or transfer appropriateness (*p* = 0.68)—both of which were significantly different from the guidelines.

**Conclusion:**

Education of primary hospital staff improved the immediate knowledge gained but did not improve record-keeping or the appropriateness of inter-hospital patient transfer.

**Trial registration:**

The study was registered with Sri Lanka Medical Associations’ clinical trial registry. Reg. No SLCTR-2013–023. Registered: 30/07/2013.

**Supplementary Information:**

The online version contains supplementary material available at 10.1186/s12909-023-04375-1.

## Introduction

### Background

Snakebite is considered a neglected tropical disease [1]. In Sri Lanka, there are approximately 40,000 hospital admissions following snakebites reported annually [2, 3]. In a nationwide community-based study that included snakebite victims who did not present to a hospital, there were more than 80,000 bites annually [4]. Most of the envenoming results from land snakes such as Russell’s viper (*Daboia russelii*), Hump-nosed pit viper (*Hypnale* species), Common krait (*Bungarus caeruleus*), Saw-scaled viper (*Echis carinatus*), and Cobra (*Naja naja*) [5–7]. In Sri Lanka, the mortality rate from snakebites was 1.91 per 1000 hospital admissions [2]. The incidence of snakebite deaths (including deaths in the community and hospital) was 2.3 per 100,000 population [4]. The amount of time it takes to get to the hospital has also decreased over the past decades, which has decreased mortality and the percentage of patients who have complications [8]. Despite early patient presentation, there are still problems with envenoming diagnosis [9] and the absence of geographically specific, minimally immunogenic antivenom for Sri Lankan snakes [10]. When administering antivenom to patients who initially show no or little signs of systemic envenoming, there are delays because of worries about adverse antivenom reactions.

Modifiable factors in hospitals that may affect care include a lack of intensive care or monitoring facilities, adherence to management guidelines, and health worker knowledge.

The majority of snakebites present to primary rural hospitals, which are staffed by non-specialist medical doctors. A sizable proportion of patients are then transferred to secondary or tertiary care hospitals where specialists are available for further care [3]. Thus, the primary care hospital plays a pivotal and early role that may alter the morbidity, mortality, and cost associated with snakebites. Previous studies of self-poisoning patients presenting to primary rural hospitals highlighted professional isolation and high rates of interhospital transfer of patients that were not medically indicated [11, 12].

The Sri Lanka Medical Association (SLMA), the apex body in medicine in Sri Lanka, has a lead role in advocacy for snakebite management, including the development of management guidelines [13], a hotline service, and liaison with the health authorities. Understanding how well these guidelines are used in primary care clinical practice is important for both patient care and subsequent guideline development.

The objective of this study was to test whether an additional systematic educational program (an educational intervention) based on SLMA national guidelines improves the knowledge and clinical practice of first-contact doctors and nurses in primary care hospitals compared with access to freely available online guidelines alone.

## Methods

### Study design and enrollment

We conducted a cluster randomized control trial in the predominantly rural Kurunegala district of the North Western Province of Sri Lanka. The district has 44 primary hospitals with inpatient facilities that were eligible and randomized to the study. The primary hospitals included four larger base hospitals with subspecialty care and 40 smaller divisional hospitals with lower resources and no specialist care. Control hospitals had free access to guidelines, while intervention hospitals received a brief educational intervention. Following the completion of the educational intervention, snakebite admissions from all enrolled hospitals were collected for one year between May 25^th^, 2013, and May 25^th^, 2014. Patients were identified from the ward admission records by clinical research staff who visited study hospitals every second and fourth week.

### Sample

All patients with a history of snakebites who presented to the 44 study hospitals for a period of one year from May 25th, 2013, to May 25th, 2014, and were included in the analysis. We anticipated that including all the snakebites admitted to all study hospitals for one year would avoid biases.

### Randomization

Each hospital was considered to be a cluster. The cluster randomization was undertaken by an independent statistician. Randomisation was stratified by including the following variables: government category of hospitals (reflecting hospital size and facilities), number of snakebite admissions, and geographical areas of the hospital in relation to Kurunegala district. There were 24 hospitals randomized to the intervention group and 20 hospitals to the control group.

### Interventions

A full-day interactive teaching session, including group discussions, lectures, role-play, and demonstrations, was conducted at a central location in the district. The workshop was designed and delivered by experts (locally known senior consultant physicians, the principal researchers, and a herpetologist) and covered topics such as identification of the offending snakes, epidemiology and manifestations of envenoming, complications, appropriate transfer referrals, appropriate use of antivenom (AV), and management of AV reactions and resuscitation as per SLMA guidelines. Anticipated ventilatory problems, the requirement for intensive care unit care, severe coagulopathy, impending acute kidney injury, the necessity for surgical procedures for local necrosis and refractory shock, a lack of resuscitation facilities, AV, and emergency medicines were the accepted indicators for transfers.

The participants' knowledge was evaluated at the beginning and end of the session using pre- and post-tests, respectively.

Participants in the intervention workshop were encouraged to share their newly acquired knowledge and skills with the rest of their treating facility's staff as soon as possible. To support this activity, the research team visited each intervention hospital within a period of one week from the day of the workshop to facilitate the intended interactive discussions between the session participants and the rest of the medical staff at their respective treating facilities. The entire management team of each hospital in the intervention group was invited for a guided discussion. The study team provided the multimedia and other facilities to convene these interactive discussions. If any staff were absent on these occasions due to their commitments, arrangements were made to brief them on the subject on another day at their convenience. The procedure was reinforced throughout the study.

Each intervention hospital received the SLMA treatment guidelines [13], a user-friendly patient medical record for snakebite admission Known as a Bed Head Ticket (BHT) folio annexures (Additional File 1_ BHT_folio), and a wall poster depicting pictures of deadly venomous snakes and essential management steps depicted in an algorithm. This was supported by promotional items such as pens and T-shirts with printed messages promoting the use of the national guidelines (Table 1). The study team ensured the availability and use of the intervention tools by the intervention hospital group throughout the study.

**Table 1:** Components of the educational Intervention

1. Interactive training workshops, based on SLMA guidelines [13], were delivered by experts in this field to workshop participants, including doctors and nurses representing all the primary hospitals in the intervention group
2. Documentation (user-friendly, preformatted, comprehensive BHT folio annexures of the admission form for snakebite patients). Introduction of additional educational wall charts with important messages based on SLMA guidelines, e.g., treatment of anaphylaxis
3. Distribution of educational materials: a poster depicting pictures of deadly venomous snakes and essential management steps in an algorithm
4. Promotional items such as pens and T- shirts with printed messages promoting the use of the national guidelines

The control group did not participate in the workshop and received neither the printed version of the freely available online SLMA treatment guidelines nor any other components of the intervention. The study team did not offer any training to the staff of the control hospitals.

### Preparation and validation of components of the intervention (at Pre-intervention period)

#### Preformatted BHT folio annexures

The pre-formatted BHT folios were initially developed by locally known senior consultant physicians and a group of medical practitioners from primary health care units. This version was trialed for snakebite admissions in a number of primary hospitals. Subsequent feedback was incorporated in the final version used for the intervention.

#### Educational charts

The initial version of the wall posters depicting pictures of deadly venomous snakes and essential management steps depicted in an algorithm was prepared by a content specialist in accordance with the guidelines of SLMA snakebite management and circulated to a group of locally known senior clinicians and a herpetologist for content verification.

#### Data collection and data management

The data extracted from the patients' hospital records were transcribed into pre-structured data extraction forms (Additional File 2_Data Extraction form) and then entered by trained research assistants into a central database in Microsoft Access. The main variables extracted were demographic information, snake identification, clinical signs, and reasons for the transfers and treatment. The patients’ records were also scanned and linked to the patient’s database record.

Prior to data analysis, all database entries were independently cross-checked with the scanned patient record by two other independent researchers who would reach consensus on any correction. The presence of any clinical feature was recorded as present if it was recorded in the structured folio or in the medical staff’s clinical notes. Clinical features not recorded as present in the Clinical feature section of the folio were coded as not present.

Data linkage was undertaken to identify the outcome of any patient transferred from a primary hospital to the Teaching Hospital, Kurunegala (THK). After data linkage for transfers, all subsequent analysis was anonymized.

The details of the hospital stocking of antivenom and the contents of the resuscitation trolley were checked routinely for the availability of bag valve masks (Ambu bags) and test tubes to conduct the 20-min Whole Blood Clotting Time (WBCT20) test, used to identify the clinically significant coagulopathy in snakebites [14].

#### Ethics statement

The study was approved by the Ethical Review Committee (ERC) of the Faculty of Medicine, University of Peradeniya, Sri Lanka. The study was conducted in collaboration with the North Western Province's treating health authorities. The individual patient's informed consent was not required by the ERC and province since they saw the data collection component of this study as part of clinical audit. All methods were carried out in accordance with relevant guidelines and regulations.

#### Outcomes and assessments

The pre- and post-tests of pre-structured multiple-choice questionnaires (MCQ) were evaluated to check the knowledge gained at the beginning and end of the teaching session, respectively.

The primary outcomes were the post-interventional improvement of documentation in the hospital records (BHT), the number of appropriate transfers, and overall patient management graded by an expert clinician who was blinded to the study randomization.

Overall patient management was comprised of the accuracy of identification of offending snakes, evidence of envenoming, indication for antivenom (AV) therapy, regimen and dose of AV therapy, and management of AV adverse reactions. The secondary outcomes of mortality at different levels and the audit of morbidity in transferred cases were evaluated at referral hospitals.

#### Expert review

An expert clinician independently reviewed and scored the scanned copies of the primary hospital records to identify the appropriateness of transfers to the tertiary care centres and overall patient management. The clinician was blind to the randomization status of the hospital. Overall patient management was assessed using 24 predefined criteria (Additional File 3_24 Predetermined_Points) based on the SLMA guideline. Criteria included basic demographic details, the history of the bite, basic clinical parameters, and specific clinical features, along with treatment and hospital outcomes. Overall patient management was assessed as satisfactory if it fulfilled the following criteria: correct identification of the type of bite, correct identification of envenomation features, clinical management of envenomation, correct indication for AV, and the identification and management of complications (of snakebite or AV). The tertiary hospital records were also reviewed for those patients who were transferred from a primary hospital. The accepted indications of transfers were anticipated ventilatory problems, the need for ICU care, severe coagulopathy, impending acute kidney injury, the need for surgical care for local necrosis and refractory shock, a lack of resuscitation facilities, and the AV and emergency medications.

### Statistical analysis

The data was analysed using the R software statistical package version 3.2.5 and the packages aod [15] and clusrank [16].

The median and IQR were calculated for pre- and post-test, and a Wilcoxon signed-rank test was performed to compare the scores between them (paired samples). The Clustered Wilcoxon rank-sum test was performed to compare the scores between the quality of the patients’ medical records between the two groups (independent samples) [17], which were assessed for 24 pre-determinant points. The test of the proportion of homogeneity for appropriate transfers, if the patient was managed correctly at primary hospitals, was performed through the Donner ICC adjusted Chi-squared test [18, 19]. The p-value of 0.05 was regarded as statistically significant.

## Results

The intervention group consisted of 22 divisional hospitals and two base hospitals. Each hospital sent two to three healthcare professionals, including first-contact physicians and nurses, to the workshop. Compared to base hospitals, which have much greater staff numbers, divisional hospitals had a higher percentage of staff attending the training, with a representation of > 30% of the hospital's clinical staff and, in some cases, up to 100% [the median number of doctors working in the divisional hospitals was 3 and nursing officers were 6].

Fifty participants from interventional hospitals participated in the training workshop. The pre-test results for the initial evaluation were available for all the participants. For the subsequent analysis, only 34 post-test results were available, as 16 participants left the workshop just before the post-test to attend to their official duties or personal commitments. However, they were present during the whole workshop, which ran into both the morning and afternoon of the day. After the workshop, Knowledge had improved significantly from a pre-test median score of 16/40 (IQR: 10.25–20) to a post-test median score of 23.5/40 (IQR: 18.25–28) (*p* < 0.00001).

Within the intervention group of hospitals, in-house education was provided by staff who attended the workshop in all but two hospitals. Those two hospitals were among the four hospitals excluded from the cluster analysis as they had no snake bite admissions. Furthermore, it was estimated that more than 90% of the staff in each hospital were engaged in the in-house interactive sessions.

After one year of follow-up, the primary analysis included 1021 snakebite cases from 20 intervention hospitals and 1165 cases from 17 control hospitals. There were 107 patients (10.5%) in the intervention group and 142 (12%) patients in the control group who reported comorbid medical conditions. The total number of co-morbidities was reported either as sole manifestations or in combination, without taking into account the differences in patient baseline characteristics of age, gender, and type of bite reported (Tables 2 and 3). There were no deaths reported from either the groups in the primary hospitals or those subsequently transferred to the tertiary care units. The details of the hospital outcomes of the patients are given in Table 4.

**Table 2:** Baseline Characteristics of Snakebite patients admitted to study Hospitals in Kurunegala District of Sri Lanka

	**Intervention** n (%)	**Control** n (%)
**Gender**		
• Male	606 (59.3)	683 (58.6)
• Female	415 (40.6)	482 (41.3)
**Age groups**		
• < 12	58 (5.7)	81 (6.9)
• 12–19	88 (8.6)	98 (8.4)
• 20–29	143 (14)	156 (13.4)
• 30–39	190 (18.6)	210 (18)
• 40–49	202 (19.8)	225 (19.3)
• > = 50	340 (33.3)	395 (33.9)
**Type of Bite**		
• Russell’s Viper	30 (2.9)	31 (2.7)
• Hump-nosed Viper	414 (40.5)	409 (35)
• Cobra	3 (0.3)	11 (1)
• Common Krait	5 (0.5)	8 (0.7)
• Saw Scaled Viper	2 (0.2)	1 (0.1)
• Green pit Viper	0	3 (0.3)
• Non venomous bite	28 (2.7)	33 (2.8)
• Unidentified bites	539 (52.8)	669 (57.4)
**Total**	**1021**	1165

**Table 3:** Baseline characteristics of co-morbidities of patients with snakebites in the study hospitals in Kurunegala district

**Type of Medical Comorbidities** ^a^	**Intervention** n (%)	**Control** n (%)
Hypertension	47 (4.6)	46 (4)
Diabetes mellitus	21 (2)	28 (2.4)
Ischemic Heart Diseases	3 (0.3)	8 (0.7)
Choric Kidney Diseases	4 (0.4)	2 (0.2)
Hyperlipidemia	3 (0.3)	4 (0.3)
Osteo/Rheumatoid Arthritis	1 (0.1)	2 (0.2)
Bronchial Asthma	15 (1.5)	25 (2)
Other lung Infection’s diseases	2 (0.2)	1 (0.1)
Known allergy to either medications or food	18 (1.8)	23 (2)
Anemia and blood disorders	2 (0.2)	4 (0.3)
Hypothyroidisms	3 (0.3)	1 (0.1)
GORD	1 (0.1)	1 (0.1)
Carcinoma (Oral, Breast)	-	2 (0.2)
Epilepsy	2 (0.1)	3 (0.3)
Past history of Snakebites	1 (0.1)	3 (0.3)
Psychiatric illness	2 (0.2)	4 (0.3)
Pregnancy/Lower segment caesarean section (LSCS)		3 (0.3)
Past surgical histories related to other medical conditions	1 (0.1)	4 (0.3)
Other medical conditions	2 (0.2)	2 (0.2)

^a^The total number of Co- morbidities were reported either as sole manifestations or in combination

**Table 4:** Hospital outcomes of snakebite patients admitted to primary Hospitals in Kurunegala District in North Western Province of Sri Lanka

Primary hospital outcomes of the snakebite admissions	Intervention	Control
	n (%)	n (%)
Discharged	680 (67)	742 (64)
Transferred	208 (20)	191 (16)
Death	0	0
Left the hospital before discharge decision and went home	133 (13)	232 (20)
**Total**	**1021**	**1165**

**Fig. 1:**
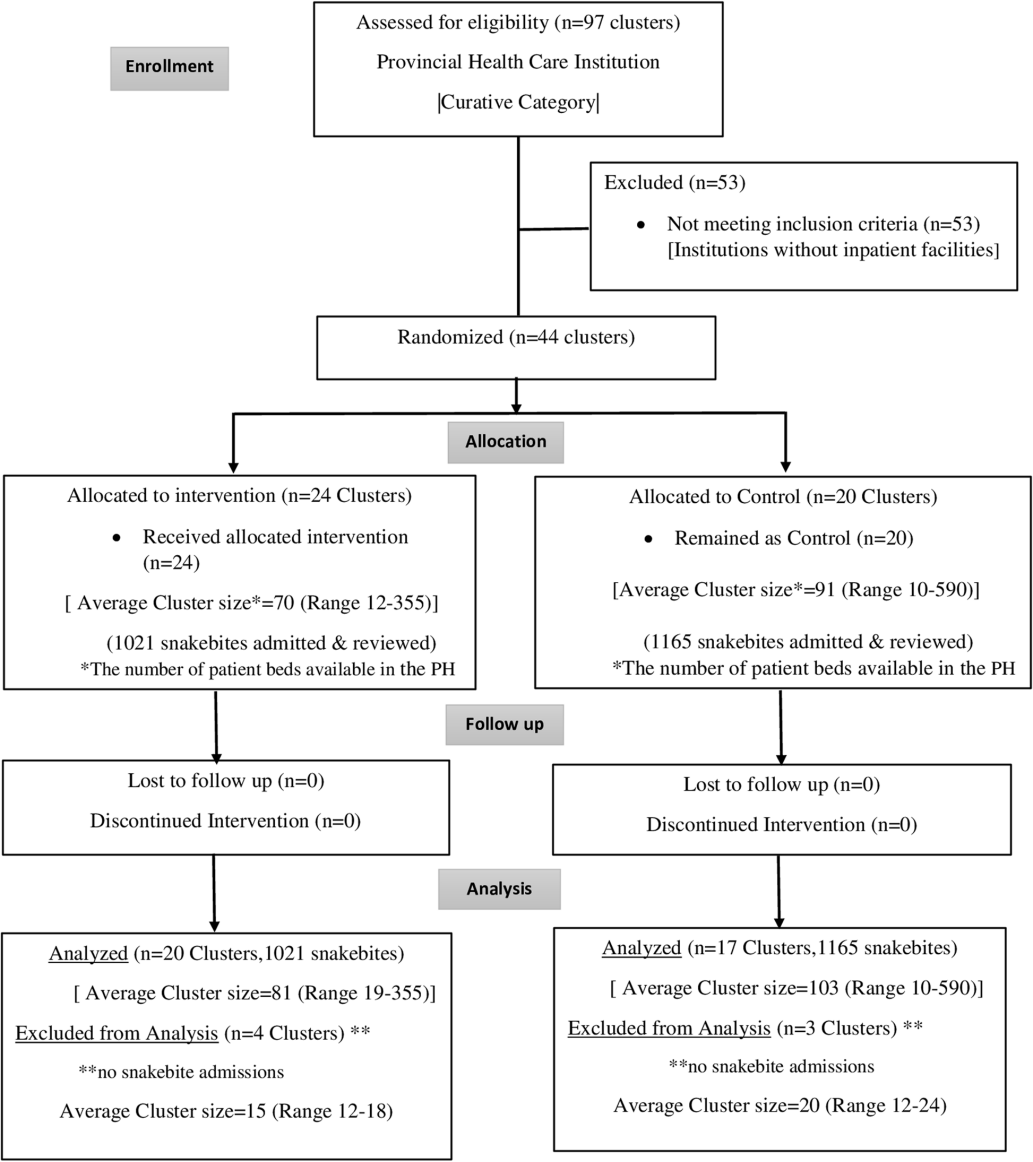
Consort flow diagram Recruitment flow chart of clusters and participants; a hospital-based educational intervention program to improve the snakebite management in Primary Hospitals

Four hospitals in the intervention group and three hospitals in the control group did not have cases of snakebite and were subsequently excluded from the cluster analysis (Fig. 1).

### Primary outcomes

**Fig. 2:**
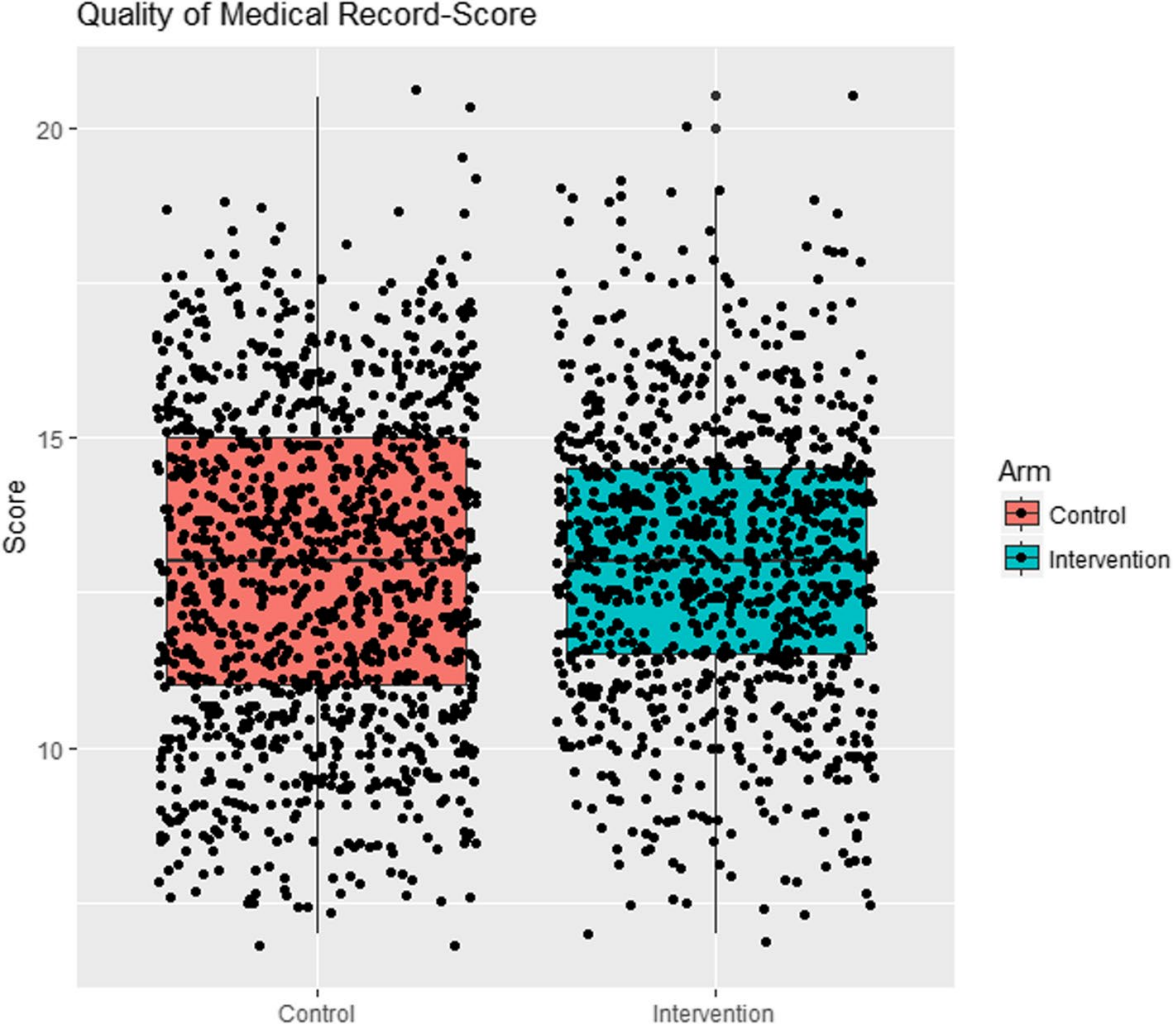
Comparison of two groups-scores for quality of recording in hospital records for 24 pre-determined points-scatter diagram

There was no difference between the groups in any of the 3 primary outcomes of improvement in the quality of patient records (Fig. 2), appropriateness of transfers, or overall patient management (Table 5). Overall clinical management was high in both groups. The quality of documentation in the BHT was poor, and rates of inappropriate transfer were high in both groups.

**Table 5:** Details of the numbers analyzed by cluster and Intraclass correlation coefficients

Primary Outcome Variables	Intervention group	Control group	ICC^a^	Adj. test stat	*p*-value
01) Quality of Medical Records					
No. Hospitals	20	17			
Snakebite patients admitted	1021	1165			
• Median scores	13 (IQR:11.5-14.5)	13 (IQR 11-15)		-0.5486^b^	0.5833
02) Appropriateness of Transfers					
No. Hospitals	18	15			
• Transfer indicated	32/208	26/191	0.0252	0.2^c^	0.6823
03) Overall patient management*					
No. Hospitals	20	17			
• Satisfactory**	785/888	852/933	0.1016	0.4^c^	0.5032
(*The patients who left the hospital against the medical advice [LAMA] were excluded from this assessment)					

^a^Intra-cluster correlation (Anova estimate)

^b^Clustered Wilcoxon rank sum test

^c^ICC Adjusted chi-squared test

*The patients who left the hospital early before discharge decisions were excluded from this assessment

**If the patient correctly managed as per SLMA guideline

**Fig. 3:**
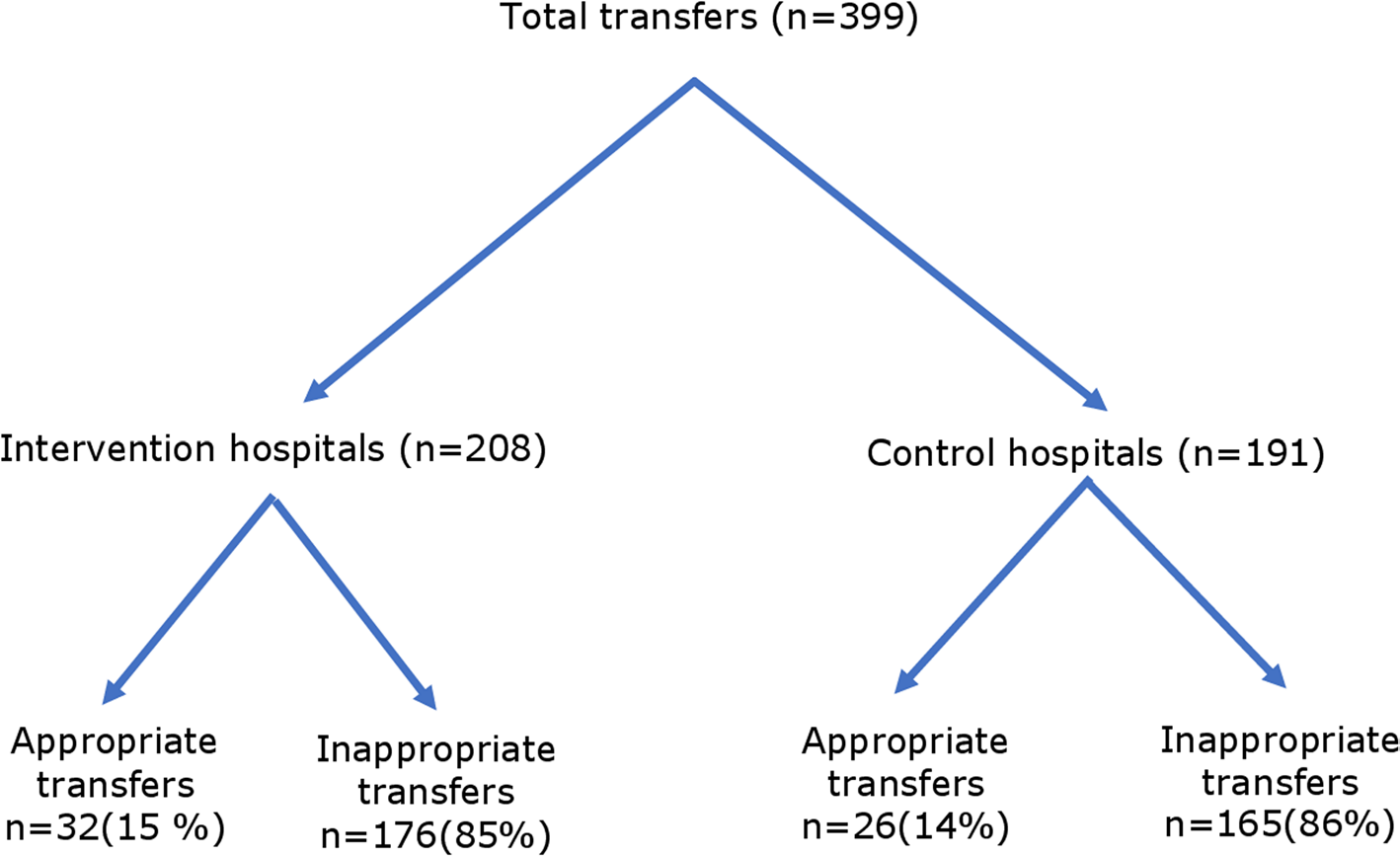
Flow chart diagram Total number of transferred patients; appropriate vs inappropriate transfers

**Fig. 4:**
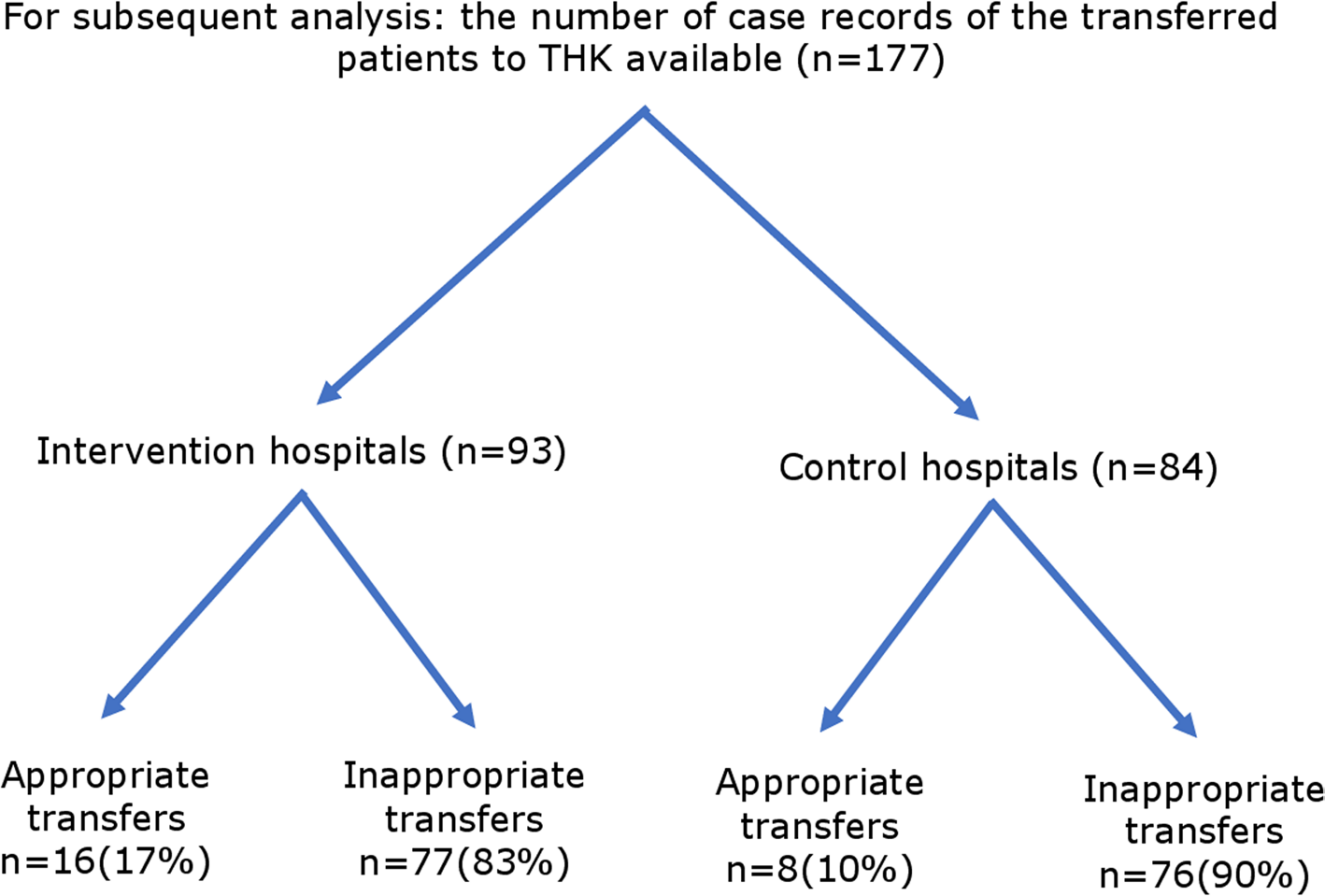
Flow chart diagram Subsequent analysis of Transferred patients to tertiary care unit Teaching Hospital Kurunegala (THK); appropriate vs inappropriate

There were 208 (20.4%) patients transferred out of the primary hospitals in the intervention group, compared with 191 (16.4%) cases in the control group. Whether the transfer decisions were appropriate or not based on records is shown in Fig. 3, with the appropriate transfers at 15% (32/208) vs. 14% (26/191), respectively. Of these, 294 cases were transferred to the district tertiary care unit of Teaching Hospital Kurunegala (THK) and 105 patients to a facility in another health district. Of the transfer cases to THK, 93 (60.4%) case records in the intervention group and 84 (60%) case records in the control group were available for subsequent analysis. A subgroup analysis of the appropriateness of the decision to transfer to THK concerning the study arm is shown in Fig. 4, with 17% vs. 10%, respectively. Of these, 30 (17%) cases received AV at THK (19 cases in the intervention group vs. 11 cases in the control group). Of them, 14 cases were identified by the expert review as having indications for AV in their respective primary hospitals (9 in the intervention group vs. 5 in the control group).

## Discussion

There were no deaths within the entire cohort. Rates of overall satisfactory treatment, including appropriate identification and transfer decisions for high-risk cases, were high in both study arms (> 90%). This suggests the combination of prior education, experience, and 10 years of SLMA national guidelines has been effective in addressing the core clinical challenges of snakebite in Sri Lanka.

Disappointingly, the educational intervention had no impact on the high rates of inappropriate inter-hospital transfers occurring without medical indication and outside of the current guidelines in both groups. Even with medically minor snakebites, the majority of patients who present to the primary hospital are subsequently transferred to territory care for further management [20]. Decisions to transfer patients may be influenced not only by medical indications but also by social pressure. In Sri Lanka, the cost of inter-hospital transfers accounts for a significant portion of total patient costs [21]. The cost of hospital transfer accounted for almost half of the total treatment costs at the primary hospital level for pesticide poisoning in Sri Lanka [22]. Given the high quality of overall management in the primary hospitals, this paradoxical result requires further investigation to refine a more effective intervention. Within our study, lack of local resources did not explain transfer rates. A previous study has shown that medical decisions in primary hospitals may be influenced both by non-medical personal and community expectations [21], in a context where doctors may feel isolated [23]. Doctors in primary care hospitals are in direct contact with the public, and their image and reputation are at stake if a death occurs under their care. Future interventions to address transfer rates should highlight the success of primary hospital management of snakebite demonstrated in this study. Such interventions may need to be directed to the local community as well as medical staff.

There was no difference in the quality of documentation between the groups. There are a number of explanations for this, and it needs to be addressed in the context of the high rates of overall clinical compliance. The first is that some of the scored items may have little clinical relevance, such as patient education and occupation details, in determining individual patient management. While structured admission forms for toxicology have been shown to improve documentation [24], there has to be a perceived benefit for the clinician in using such forms. Such a benefit may not be perceived by an individual doctor working remotely who is not providing clinical information to others outside of the primary hospital.

As over 80% of snakebites in Sri Lanka are treated in primary hospitals [3], it is important to ensure that management is of high quality and designed to be delivered within these clinical settings. The current results support the fact that this is being achieved. For many years, there has been relatively little change in snakebite treatment in Sri Lanka, but it is important to have mechanisms that can effectively propagate clinical change if it occurs. The most likely change in Sri Lanka will be the introduction of new antivenoms with different doses and indications for use. Brief educational interventions have previously been successful in promoting guideline change in Sri Lanka [25], and further research on how to disseminate training to small rural institutions needs to be done.

### Limitation

As with much research based on chart reviews, the list of basic demographic details and some clinical data, especially in mild cases, is probably not complete. However, it is unlikely to influence the treatment decision linked to the study's findings.

Anecdotally, we also became aware that some educational materials had filtered to control group hospitals due to possible transfers of the medical staff within the study district that might have taken place during the period of the study and that SLMA hotline services on advocacy for snakebite management might be utilized by the treating physicians at control group hospitals. Given the high rate of satisfactory care in both groups, it is unlikely that a clinically significant improvement in care could be demonstrated.

## Conclusion

The study, while improving knowledge, did not improve the overall quality of snakebite treatment, which was high in both control and intervention hospitals. The rates of inappropriate inter-hospital transfers were high and not improved by the intervention. Given the cost of an inter-hospital transfer, the determinant of the decision to transfer requires further research.

## Supplementary Information

**Additional file 1: **BHT_folio.

**Additional file 2: **Snake Bite Patient Data Entry Form- Peripheral Hos pital.

**Additional file 3: **24 Predetermined Points.

**Additional file 4: **Dataset_RCTscore.

**Additional file 5: **Dataset_Transfer.

**Additional file 6: **Dataset_ Managed.

**Additional file 7: **Formulas for R script.

## Data Availability

All data generated or analysed during this study are included in this published article (and its Additional supplementary Information files).

## Abbreviations

Ambu bags: Bag Valve Mask
AV: Antivenom
BHT: Bed Head Ticket
ICC: Intraclass Correlation Coefficient
LAMA: Left Against Medical Advice
NWP: North Western Province of Sri Lanka
IRB: Institutional Review Board
MCQ: Multiple Choice Questions
SLMA: Sri Lanka Medical Association
THK: Teaching Hospital Kurunegala
WBCT20: 20 Minute Whole Blood Clotting Time

## References

[1] Warrell DA. Guidelines for the management of snake-bites. Guidelines for the management of snake-bites. 2010.

[2] Lanka MoHS. Annual Health Bulletin. In: Unit MS, editor. http://www.health.gov.lk/moh_final/english/public/elfinder/files/publications/AHB/2020/AHB_2018.pdf: Ministry of Health , Government of Sri Lanka; 2018. p. 247.

[3] Shahmy S, Kularatne SA, Rathnayake SS, Dawson AH (2017). A prospective cohort study of the effectiveness of the primary hospital management of all snakebites in Kurunegala district of Sri Lanka. PLoS Negl Trop Dis.

[4] Ediriweera DS, Kasturiratne A, Pathmeswaran A, Gunawardena NK, Wijayawickrama BA, Jayamanne SF (2016). Mapping the risk of snakebite in Sri Lanka-a national survey with geospatial analysis. PLoS Negl Trop Dis.

[5] Alirol E, Sharma SK, Bawaskar HS, Kuch U, Chappuis F (2010). Snake bite in South Asia: a review. PLoS Negl Trop Dis.

[6] Kasturiratne A, Pathmeswaran A, Fonseka M, Lalloo D, Brooker S, de Silva H. Estimates of disease burden due to snakebite in Sri Lankan hospitals. 2003. 16124448

[7] Kularatne S (2001). Clinical profile of snake envenoming: a study in North Central Province of Sri Lanka. The 23rd bibile memorial oration in 2001. Sri Lanka J Med.

[8] Silva A, Hlusicka J, Siribaddana N, Waiddyanatha S, Pilapitiya S, Weerawansa P, et al. Time delays in treatment of snakebite patients in rural Sri Lanka and the need for rapid diagnostic tests. PLoS neglected tropical diseases. 2020;14(11):e0008914. 10.1371/journal.pntd.0008914 PMC7728389 33253208

[9] Isbister G, Maduwage K, Shahmy S, Mohamed F, Abeysinghe C, Karunathilake H (2013). Diagnostic 20-min whole blood clotting test in Russell’s viper envenoming delays antivenom administration. QJM.

[10] Keyler D, Gawarammana I, Gutiérrez JM, Sellahewa K, McWhorter K, Malleappah RJT. Antivenom for snakebite envenoming in Sri Lanka: the need for geographically specific antivenom and improved efficacy. Toxicon. 2013;69:90–7. 10.1016/j.toxicon.2013.01.022 23454626

[11] Senarathna L, Hunter C, Dawson AH, Dibley MJJQhr. Social dynamics in rural Sri Lankan hospitals: revelations from self-poisoning cases. Qualitative health research. 2013;23(11):1481–94. 10.1177/1049732313510361 24135311

[12] Senarathna L, Buckley NA, Dibley MJ, Kelly PJ, Jayamanna SF, Gawarammana IB, et al. Effect of a brief outreach educational intervention on the translation of acute poisoning treatment guidelines to practice in rural Sri Lankan hospitals: a cluster randomized controlled trial. PloS one. 2013;8(8):e71787. 10.1371/journal.pone.0071787 PMC3747188 23990989

[13] Association ECoSSLM. GUIDLINES FOR THE MANAGEMENT OF SNAKEBITE IN HOSPITAL SLMA Colombo 07: SLMA; 2013 . Electronic Guidlines Vesrion 3.0:2013 revised and expanded: Available from: http://slma.lk/wp-content/uploads/2014/12/Guidelines-for-management-of-snake-bite-in-hospital.pdf. Updated March 2013; cited 2013.

[14] Ratnayake I, Shihana F, Dissanayake DM, Buckley NA, Maduwage K, Isbister GK (2017). Performance of the 20-minute whole blood clotting test in detecting venom induced consumption coagulopathy from Russell’s viper (Daboia russelii) bites. Thromb Haemost.

[15] Lesnoff M, Lancelot, R. aod: Analysis of overdispersed data. R package version. 2012;1(1).

[16] clusrank: Wilcoxon Rank Sum Test for Clustered . Data. R package version 0.5–2. 2017. Available from: https://CRAN.R-project.org/package=clusrank.

[17] Rosner B, Glynn RJ, Lee MLT (2006). Extension of the Rank Sum Test for Clustered Data: Two-Group Comparisons with Group Membership Defined at the Subunit Level. Biometrics.

[18] Donner A. Statistical methods in ophthalmology: an adjusted chi-square approach. Biometrics. 1989:605-11. 2765640

[19] Jeong KM (2016). Tests for homogeneity of proportions in clustered binomial data. Commun Stat Appl Methods.

[20] Thalgaspitiya S, Isbister G, Ukuwela K, Sarathchandra C, Senanayake H, Lokunarangoda N, et al. Bites by snakes of lesser medical importance in a cohort of snakebite patients from rural Sri Lanka. Toxicon. 2020;187:105–10. 10.1016/j.toxicon.2020.08.025 32891665

[21] Wickramasinghe K, Steele P, Dawson A, Dharmaratne D, Gunawardena A, Senarathna L (2009). Cost to government health-care services of treating acute self-poisonings in a rural district in Sri Lanka. Bull World Health Organ.

[22] Ahrensberg H, Madsen LB, Pearson M, Weerasinghe M, Eddleston M, Jayamanne S, et al. Estimating the government health-care costs of treating pesticide poisoned and pesticide self-poisoned patients in Sri Lanka. Global health action. 2019;12(1):1692616. 10.1080/16549716.2019.1692616 PMC6896413 31775583

[23] Senarathna L, Hunter C, Dawson AH, Dibley MJ (2013). Social dynamics in rural Sri Lankan hospitals: Revelations from self-poisoning cases. Qual Health Res.

[24] Buckler NA, Whyte IM, Dawson AH, Reith DA (1999). Preformatted admission charts for poisoning admissions facilitate clinical assessment and research. Ann Emerg Med.

[25] Senarathna L, Buckley NA, Dibley MJ, Kelly PJ, Jayamanna SF, Gawarammana IB (2013). Effect of a brief outreach educational intervention on the translation of acute poisoning treatment guidelines to practice in rural Sri Lankan hospitals: a cluster randomized controlled trial. PLoS ONE.

