# Supplementary material for: Compliance with national snakebite treatment guidelines in rural Sri Lankan hospitals: a cluster randomized controlled trial of a brief educational intervention
Source: BMC Med Educ. 2023 May 27;23:390. doi: 10.1186/s12909-023-04375-1 (PMC10225084; doi:10.1186/s12909-023-04375-1)
Supplement: Supplementary file 1 — Additional file 1: BHT_folio. [file 12909_2023_4375_MOESM1_ESM.pdf]

BHT Folio for Snakebite Patients - Rapid Assessment

Details of Snakebite

Date of bite: **Time of Bite:** ……………am/pm

Place of bite: Home ☐ Paddy field ☐ Chena ☐ Road side ☐ Estate ☐
Jungle ☐ Other (specify)…………………………………………….

Part of body bitten: **Right** ☐ **Left** ☐

Toes ☐ Foot ☐ Ankle ☐ Leg ☐ Thigh ☐
Fingers ☐ Hand ☐ Forearm ☐ Arm ☐

Other (specify)……………………………………………………………………

Unnoticed bite: ……

……

Medical / Surgical History of Patient

Co-morbidities:

……

……

Medication:

……

……

Allergic History:

……

……

(If an in transfer)

Indication for transfer:

……

……

Treatment given at local hospital:

……

……

……

……

……

Identification of offending snake

Snake examined and identified by doctor **in this** hospital

If Yes:

Specimen identification

Russell's viper ☐ Cobra ☐ Hump-nosed viper ☐ Common krait ☐ Saw-scaled viper ☐

Other venomous…………………………………………………….

Other non-venomous ……………………………………………….

If No:

Snake examined and identified by doctor in another hospital

If yes:

What is the identification………………………………………………………………………………………………

If no:

Snake was identified only by patient / observer using photo match:

If So, identity of the snake…………………………………………………………………………………………………

Identification based on history/ observation

If So, identity of the snake ………………………………………………………………………………………………….

Patient's activities at the time of bite

Harvesting ☐ Cultivation ☐ Sleeping ☐ Walking ☐

Other (specify)……………………………….

First aids given

…….

…….

…….

…….

…….

BHT Folio for Snakebite Patients - Investigations

WBCT- 20(Whole Blood Clotting Test):

Number of test	Date	Time	Reading
1st			
2nd			
3rd			
4th			

Other Investigations and results (Include the date / time):

1. ………

2. ………

3. ………

4. ………

5. ………

6. ………

7. ………

8. ………

BHT Folio for Snakebite Patients – Monitoring

**Clinical features:**	**Time/Date of Assessments**							
	**On Adm.**							
**General:**								
Abdominal pain								
Chest pain								
Vomiting								
Difficulty in breathing								
Headache								
Muscle cramps								
Myalgia								
Other								
**Local:**								
Pain								
Fang marks								
Swelling								
Blistering								
Necrosis								
Discolouration								
**Coagulopathy:**								
Bleeding at bite - site								
Mucosal bleeding								
GI - bleeding								
**Neurotoxicity:**								
Ptosis								
Double vision								
Eye muscle weakness								
Weak neck flexion								
Limb muscle weakness								
Dysphagia								
Loss of consciousness (N/D/C/SC/UC)								
**Abdomen:**								
Abdo (N/soft/distended)								
Bowel sounds(+/-)								

**Essential monitoring**								
Pulse rate								
Blood pressure								
Respiratory rate								
Tidal volume								
Urine output								
Input								

BHT Folio for Snakebite Patients – Antivenom Treatment

(Please Fill Separate Forms For Each Episode of Antivenom Infusion)

Date/Time:

Indication/s for antivenom:

Neurotoxicity ☐ Coagulopathy ☐ Local effects ☐

other (specify)…………………………………………

Number of vials: ………………………………………..

Manufacturer / Batch No of vials:

………

Body Weight: (If a child)

Fluid Volume: ………………………………

………

Date/Time of Starting Infusion:

………

Reaction to antivenom:

No reaction ☐ Mild reaction ☐ Moderate reaction ☐ Severe reaction ☐

Mild reaction: (Only cutaneous allergy)
Moderate reaction: (Mild drop of blood pressure and bronchospasm)
Severe reaction: (Severe anaphylaxis with low BP and bronchospasm)

Management of reaction:

………

………

………

………

Date/Time of Completing/Terminating the Infusion:

………

………

BHT Folio for Snakebite Patients – Data Collection

Patient's details:

Pre-School ☐ Schooling ☐ Higher-Education ☐ Unemployed ☐ Farmer (Fulltime) ☐

Farmer (Part Time) ☐ Other (specify)……………………………………

Level of education:

Grade <5 ☐ Grade 5-11 ☐ AL ☐ Tertiary education ☐

Outcome/ Remarks:

Recovered ☐ Date of discharge …………………………………..

Transfer ☐ Date/Time ………………………………………………

Reason ……………………………………………………………………………………………………….

…….

Death ☐ Date/Time ………………………………………………

Cause of Death ………………………………………………………………………………………
