# Supplementary material for: Compliance with national snakebite treatment guidelines in rural Sri Lankan hospitals: a cluster randomized controlled trial of a brief educational intervention
Source: BMC Med Educ. 2023 May 27;23:390. doi: 10.1186/s12909-023-04375-1 (PMC10225084; doi:10.1186/s12909-023-04375-1)
Supplement: Supplementary file 2 — Additional file 2: Snake Bite Patient Data Entry Form- Peripheral Hos pital. [file 12909_2023_4375_MOESM2_ESM.pdf]

Snake Bite Patient Data Entry Form - Peripheral Hospital

**BHT Number :**		**Date :**	
**Patient Name :**			
**Age :**		**Sex :**	Male / Female
**Creature Observed :**	Yes / No	**Weight :**	
**Creature Identified :**	Yes / No		

Type of Snake :

R viper	HN viper	SL krait	Common krait	Cobra	Other

**Snake Brought to Hospital :**			
**Length of the Snake :**			
**Bitten Date :**		Bitten Time :	
**Bite Site :**			
**Admission Date :**		Admission Time :	

First Aid Given :

Bandage	Paththu	Bite site washed with soap
Given Paracetamol		Jewelry removed from the bite site
Other..		

On Admission Examinations

**BP**		**Pulse Comments**	
**Pupils**		**RR**	
**Clotting Test**			

Details :

**Patient Conscious?**	Yes	No	Not Rec	**GCS**			
**Sweating?**	Yes	No	Not Rec	**Diarrhea**	Yes	No	Not Rec
**Vomiting?**	Yes	No	Not Rec	**Weakness**	Yes	No	Not Rec

Hemorrhage

Yes	No	Not Rec
Bleeding from bite site	Bleeding from Cannuala Site	Hematuria
Gum bleeding	Bleeding in to Mucosa	Bleedingfrom any othersite

Neurological Findings

Ptosis	Opthalmoplegia	Neck weakness	Other

Treatments Given

**IV Line Inserted**	Yes	No	Not Rec	**IV Fluids Given**	Yes	No	Not Rec
**Hydrocortisone (pre-med)**	Yes	No	Not Rec				
**Adrenaline (pre-med)**	Yes	No	Not Rec				
**AVS**	Bolus	Infusion	Not Given	**Amount**			
**Normal Saline**	Bolus	Infusion	Not Given	**Amount**			
**Dextrose**	Bolus	Infusion	Not Given	**Amount**			
**Other IV Fluids**	Bolus	Infusion	Not Given	**Amount**			

Other Treatments

Reactions to Anti venom | Yes | No |

Type of the Reaction

Treatments Given

Outcome

Death	Date:	Time:
Discharged	Date:	Time:
Transferred	Hospital:	
	Date:	Time:
