# Supplementary material for: Compliance with national snakebite treatment guidelines in rural Sri Lankan hospitals: a cluster randomized controlled trial of a brief educational intervention
Source: BMC Med Educ. 2023 May 27;23:390. doi: 10.1186/s12909-023-04375-1 (PMC10225084; doi:10.1186/s12909-023-04375-1)
Supplement: Supplementary file 3 — Additional file 3: 24 Predetermined Points. [file 12909_2023_4375_MOESM3_ESM.pdf]

24 Predetermined Points

Key Data elements to be looked in the BHTs

01) Age
02) Gender
03) Education & Occupation
04) Date & time of Bite
05) Admission date and time
06) Place of bite & Patient activity at the time of bite
07) Part of body bitten
08) Snake identification
09) Bite mark
10) Comorbidities
11) First aid details
12) Initial WBCT record
13) Essential Clinical monitoring
14) Fluid input/output chart
15) IV line inserted/not
16) General clinical Features
17) Local envenomation features
18) Systemic Clinical features
19) AVS treatment
20) Other treatment records (Premedication, other treatments)
21) Anaphylaxis reactions
22) Hospital outcome
23) Hospital outcome date
24) If transferred, indication for Transfer
