# Supplementary material for: Compliance with national snakebite treatment guidelines in rural Sri Lankan hospitals: a cluster randomized controlled trial of a brief educational intervention
Source: BMC Med Educ. 2023 May 27;23:390. doi: 10.1186/s12909-023-04375-1 (PMC10225084; doi:10.1186/s12909-023-04375-1)
Supplement: Supplementary file 7 — Additional file 7: Formulas for R script. [file 12909_2023_4375_MOESM7_ESM.docx]

Formulas for R script:

1. library(clusrank)

> clusWilcox.test(RecordScore~Arm+cluster(HospitalID),data=RCTScore)

1. > library(aod)

> donner(formula = cbind(TransferCorrect, TotalTransfer - TransferCorrect) ~ Arm, data = Transfer)

1. > library(aod)

>donner(formula = cbind(ManagedSatis, TotalManaged - ManagedSatis) ~ Arm, data = managed)
